# Expression of glioma-associated oncogene 2 (Gli 2) is correlated with poor prognosis in patients with hepatocellular carcinoma undergoing hepatectomy

**DOI:** 10.1186/1477-7819-11-25

**Published:** 2013-01-29

**Authors:** Dawei Zhang, Liangqi Cao, Yue Li, Haiwu Lu, Xuewei Yang, Ping Xue

**Affiliations:** 1 Department of Hepatobiliary Surgery, the Second Affiliated Hospital of Guangzhou Medical College, No. 250, East Changgang Road, Guangzhou 510260, China

**Keywords:** Gli2, Hepatocellular carcinoma, Prognosis, Epithelial-to-mesenchymal transition

## Abstract

**Background:**

Our previous studies showed that glioma-associated oncogene (Gli)2 plays an important role in the proliferation and apoptosis resistance of hepatocellular carcinoma (HCC) cells. The aim of this study was to explore the clinical significance of Gli2 expression in HCC.

**Methods:**

Expression of Gli2 protein was detected in samples from 68 paired HCC samples, the corresponding paraneoplastic liver tissues, and 20 normal liver tissues using immunohistochemistry. Correlation of the immunohistochemistry results with clinicopathologic parameters, prognosis, and the expression of E-cadherin, N-cadherin, and vimentin were analyzed.

**Results:**

Immunohistochemical staining showed high levels of Gli2 protein expression in HCC, compared with paraneoplastic and normal liver tissues (*P* < 0.05). This high expression level of Gli2 was significantly associated with tumor differentiation, encapsulation, vascular invasion, early recurrence, and intra-hepatic metastasis (*P* < 0.05). There was a significantly negative correlation between Gli2 and E-cadherin expression (*r* = −0.302, *P* < 0.05) and a significantly positive correlation between expression of Gli2 and expression of vimentin (*r* = −0.468, *P* < 0.05) and N-cadherin (r = −0.505, *P* < 0.05). Kaplan-Meier analysis showed that patients with overexpressed Gli2 had significantly shorter overall survival and disease-free survival times (*P* < 0.05). Multivariate analysis suggested that the level of Gli2 expression was an independent prognostic factor for HCC.

**Conclusions:**

Expression of Gli2 is high in HCC tissue, and is associated with poor prognosis in patients with HCC after hepatectomy.

## Background

Hepatocellular carcinoma (HCC) is the fifth most common malignancy and the third most common cause of death from cancer worldwide [1,2]. There are an estimated 626,000 to 1,000,000 new cases annually worldwide, with about half of these occurring in China alone [3,4]. Despite advances in surgical and chemotherapeutic approaches, the survival rate of patients with HCC is as low as 20 to 50% at 5 years, even in early-stage HCC after radical resection [5,6]. Recurrence after treatment remains one of the most important causes of poor long-term survival. Prediction of tumor carcinogenesis using molecular prognostic markers might aid in developing more effective therapeutic strategies and therefore result in better prognosis. However, to date, no identified molecular marker has shown unequivocal prognostic utility in HCC.

The Hedgehog (Hh) signaling pathway regulates body patterning, cell differentiation, and proliferation during embryonic development [7,8]. In humans, the Hh signaling pathway consists of three ligands: Shh, Ihh, and Dhh, which can bind to the transmembrane receptor Patched 1 (Ptch1). Upon ligand binding to Ptch1, Smoothened (Smo) is released, signals are transduced to the nucleus, and these converge on three Gli zinc finger transcription factors: Gli1, Gli2, and Gli3 [9]. Aberrant activation of the Hh pathway has recently been reported in various human cancers, including basal cell carcinoma, gastrointestinal malignancies, and breast, prostate, pancreatic, and lung cancers [10-16]. In addition, the Hh pathway cascade cross-talks with the WNT, EGF/FGF, and TGF-β/Activin/Nodal/BMP signaling cascades, which are implicated in epithelial-to-mesenchymal transition (EMT) through repression of E-cadherin and activation of N-cadherin, therefore, the Hh pathway is associated with invasion and metastasis of tumors [17].

Previous studies have shown that components of the Hh pathway are indicators for poor survival in bladder cancer, oral and esophageal squamous cell carcinoma, and ovarian, colon and breast cancers [18-23]. Of the three Gli transcriptional factors, Gli2 is a strong positive activator of downstream target genes, and it can induce Gli1 expression independent of Hh signaling [24]. The Gli2 protein has been reported to have high expression levels in HCC cell lines and human HCC tissues [25-28]. Our previous work showed that small hairpin (sh)RNA-mediated silencing of the Gli2 gene inhibits proliferation by inducing cell-cycle arrest at G1 phase in the HCC SMMC-7721 cell line. Moreover, knockdown of Gli2 enhanced SMMC-7721 to tumor necrosis factor (TNF)-related apoptosis-inducing ligand (TRAIL)-induced cell apoptosis via downregulation of c-FLIP and Bcl-2, consequently leading to induction of caspase-8 or caspase-9 dependent apoptosis pathway [29]. However, there have been few clinical reports investigating the relationship between Gli2 protein expression and the postoperative survival of patients with HCC, or whether Gli2 is realated to induction of EMT.

In the current study, we examined expression of Gli2 protein in HCC tissue using immunohistochemistry. We also evaluated the relationship of this expression with the clinical characteristics, expression of E-cadherin, N-cadherin, and vimentin, and prognosis to determine whether the level of Gli2 expression could be used to predict prognosis in patients with HCC after radical hepatectomy.

## Methods

### Ethics approval

The study was approved by the Ethics Committee of the Second Affiliated Hospital of Guangzhou Medical College with the following reference number: GY20080216302. Each patient provided written informed consent before hepatectomy.

### Patients and liver specimens

Samples of primary HCC tissues (n = 68) and corresponding paraneoplastic liver tissue (PLT, taken at 20 mm distance from the tumor margin) were obtained from patients (53 men, 15 women, median age 55 years; range 33–78 years) who underwent curative resection at the Second Affiliated Hospital of Guangzhou Medical College (Guangzhou, China) between February 2004 and July 2007. The diagnosis was confirmed by histologic examination. Curative resection was defined as removal of all recognizable tumor tissue with a clear microscopic margin. Specimens were obtained immediately after surgical resection. The patients were not pretreated with radiotherapy or chemotherapy before surgery. The control tissues were 20 samples of normal liver tissue (NLT), which were acquired from patients who had undergone surgery for liver trauma. All specimens were fixed in 10% formalin, and embedded in paraffin wax for immunohistochemical analysis.

The clinicopathologic variables are shown in Table 1. Tumor size ranged from 32 to 193 mm, with a median of 65 mm. The TNM (tumor, node, metastasis) stage was determined using the American Joint Committee on Cancer/International Union Against Cancer tumor classification system, with 45 patients classified as I or II and 23 as III or IV. Tumor differentiation was classified as follows: 16 tumors were well differentiated, 39 were moderately differentiated, and 13 cases were poorly differentiated.

**Table 1:** Clinicopathologic characteristics of 68 patients with hepatocellular carcinoma (HCC)

**Variable**	**Patients, n**
Gender	
Male	53
Female	15
Age, years	
≤60	43
>60	25
Virus infection	
HBV	58
HCV	2
None	8
AFP, ng/ml	
≤200	28
>200	40
Cirrhosis	
Present	48
Absent	20
Tumor size, mm	
≤50	18
>50	50
TNM stage	
I	12
II	33
III	20
IV	3
Tumor differentiation	
Well	16
Moderate	39
Poor	13
Tumor number	
Solitary	54
Multiple	14
Tumor encapsulation	
Intact	39
Absent or not intact	29
Vascular invasion	
Present	29
Absent	39

Abbreviations: AFP, alpha fetoprotein; HBV, hepatitis B virus; HCV, hepatitis C virus; TMN, tumor, node, metastasis.

### Immunohistochemistry

Formalin-fixed, paraffin wax-embedded tissue specimens were obtained. Serial sections 4 μm thick were prepared from each sample, then some sections were stained with hematoxylin and eosin for histologic diagnoses of tumor and non-tumor, while other sections were stained for Gli2 using the streptavidin–biotin horseradish peroxidase complex method. For the latter, the tissue sections were dewaxed, rehydrated with a xylene and graded alcohol series, and then incubated in 3% hydrogen peroxide for 10 minutes to block endogenous peroxidase activity. Optimal antigen retrieval was carried out in citrate buffer (pH 6.0) for 10 minutes in a microwave oven to enhance the immunoreactivity, and then sections were incubated in 10% blocking serum for 30 minutes at 37°C to reduce nonspecific binding. Primary anti-Gli2 polyclonal antibodies (Santa Cruz Biotechnology Inc., Santa Cruz, CA, USA) were diluted 1:100, and incubated with the sections at 4°C overnight. Subsequently, the secondary antibodies (biotinylated goat anti-rabbit immunoglobulin) and streptavidin peroxidase complex reagent were applied. Finally, the visualization signal was developed with diaminobenzidine (DAB), and the slides were counterstained with hematoxylin. Two investigators blinded to the clinical information evaluated each stained section.

The Gli2 reaction for Gli2 was graded according to the staining intensity (0, 1+, 2+, and 3+). The percentages of Gli2–positive cells were also scored on a four-point scale as 0 (0%), 1 (1 to 33%), 2 (34 to 66%), and 3 (67 to 100%). The sum of the intensity and percentage scores was used as the final Gli2 protein staining score. The staining pattern was defined as follows: 0, negative; 1 to 2, weak; 3 to 4, moderate; 5 to 6, strong [30,31]. For statistical analyses, scores of 0 to 2 were considered ‘low expression’ and scores of 3 to 6 were considered ‘high expression’.

### Follow-up

The follow-up period was defined as the interval from the date of operation to that of the last visit or the patient’s death. Deaths from other causes were treated as censored cases. After discharge, patients were followed up every 3 months during the first 2 years and every 6 months thereafter by clinical examination, including measurement of alpha fetoprotein, ultrasonography and computed tomography (CT). Times of tumor recurrence were recorded, based on the time of recurrences from the date of hepatectomy, they were classified as early (≤1 year) and late (>1 year) recurrences. Patients who developed recurrence were treated with radiofrequency ablation, percutaneous ethanol injection, or transcatheter arterial chemoembolization, or with re-resection when necessary. Disease-free survival (DFS) was defined as the interval from the operation date to recurrence, and overall survival (OS) was defined as the interval between the operation date and death. Follow-up was performed using email and telephone.

### Statistical analysis

The χ^2^ text or Fisher’s exact test was used to analyze the relationship between Gli2 expression and the clinicopathologic characteristics. DFS and OS were calculated using the Kaplan-Meier method, and differences were assessed using the log-rank test. The Cox proportional hazard regression model was used to examine associations between the various prognostic factors and survival. For all tests, a value of *P* < 0.05 was considered significant. All statistics were calculated using SPSS software (version 16.0; SPSS Inc., Chicago, IL, USA).

## Results

### Expression of Gli2

Follow-up data were obtained for all 68 patients. All patients were followed up until November 2010. The follow-up time ranged from 6 to 79 months, with a median follow-up time of 52 months.

**Figure 1:**
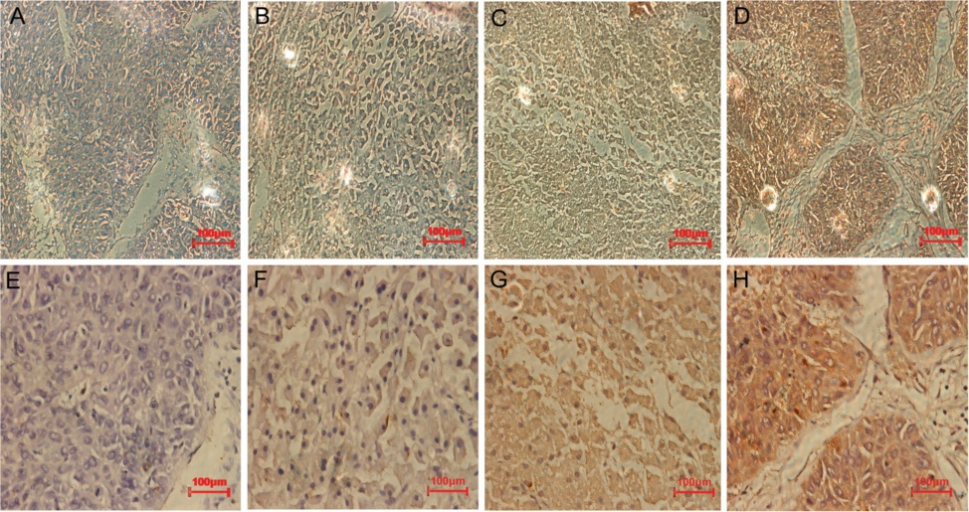
**Representative images of Gli2 immunohistochemical staining in human normal liver tissue (NLT), paraneoplastic liver tissue (PLT), and HCC.** (**A,E**) NLT with negative Gli2 expression. (**B,F**) PLT with week Gli2 expression. (**C,G**) HCC with moderate Gli2 expression. (**D,H**) HCC with strong Gli2 expression. Original magnification (A-D) × 200 for E-H × 400.

The immunohistochemical staining identified the Gli2 location in the cytoplasm and/or nucleus of tumor cells, mainly in the cytoplasm. Gli2 expression in NLT from hemangiomas and PLT were negative or weakly positive (Figure 1A,B,E,F), and mainly moderately or strongly positive in HCC (Figure 1C,D,G,H). Overall, high expression of Gli2 was recorded in 63.2% (43/68), 11.8% (8/68), and 10% (2/20) of the biopsies in HCC, PLT, and NLT samples, respectively. There were significant differences for Gli2 staining between NL and HCC (*P*<0.05), and between PLT and HCC (*P*<0.05), but there was no significant difference between NL and PLT (*P*>0.05).

### Immunohistochemistry analysis of Gli2 expression and its relationship with clinicopathologic characteristics

Correlation between the expression levels of Gli2 in HCC and various clinicopathologic variables are summarized in Table 2. A high expression level for Gli2 protein was significantly correlated with tumor differentiation, encapsulation, vascular invasion, early recurrence, and intra-hepatic metastasis (*P*<0.05). There was no significant correlation between Gli2 expression and gender, age, hepatitis virus serology, AFP level, cirrhosis, tumor size, TNM stage, or number of tumors (*P*>0.05).

**Table 2:** Correlation between Gli2 expression and clinicopathologic characteristics in HCC

**Characteristics**	**Gli2 expression**		**χ**^**2**^	***P *value**
	**High (n=43)**	**Low (n=25)**		
Gender				
Male	33	20	0.097	0.755
Female	10	5		
Age, years				
≤60	29	14	0.890	0.345
>60	14	11		
Virus infection				
HBV	35	23	0.698	0.403
HCV	2	0		
None	6	2		
AFP (ng/ml)				
≤200	20	8	1.374	0.241
>200	23	17		
Cirrhosis				
Present	30	18	0.038	0.846
Absent	13	7		
Tumor size, mm				
≤50	26	13	0.837	0.360
>50	15	12		
TNM stage				
I or II	25	20	3.375	0.066
III or IV	18	5		
Tumor differentiation				
Well	6	10	6.591	0.037 ^*^
Moderate	28	11		
Poor	9	4		
Tumor number				
Solitary	32	22	1.784	0.182
Multiple	11	3		
Tumor encapsulation				
Intact	11	15	7.930	0.005 ^*^
Absent or not intact	32	10		
Vascular invasion				
Present	23	6	5.620	0.018 ^*^
Absent	20	19		
Early recurrence				
Yes	25	8	4.324	0.038 ^*^
No	18	17		
Intra-hepatic metastasis				
Present	16	5	4.997	0.025 ^*^
Absent	17	20		

Variables are presented as number and tested by χ^2^ or Fisher’s exact test.

* *P* < 0.05.

### Association between E-cadherin, N-cadherin, vimentin, and Gli2 expression in hepatocellular carcinoma

**Figure 2:**
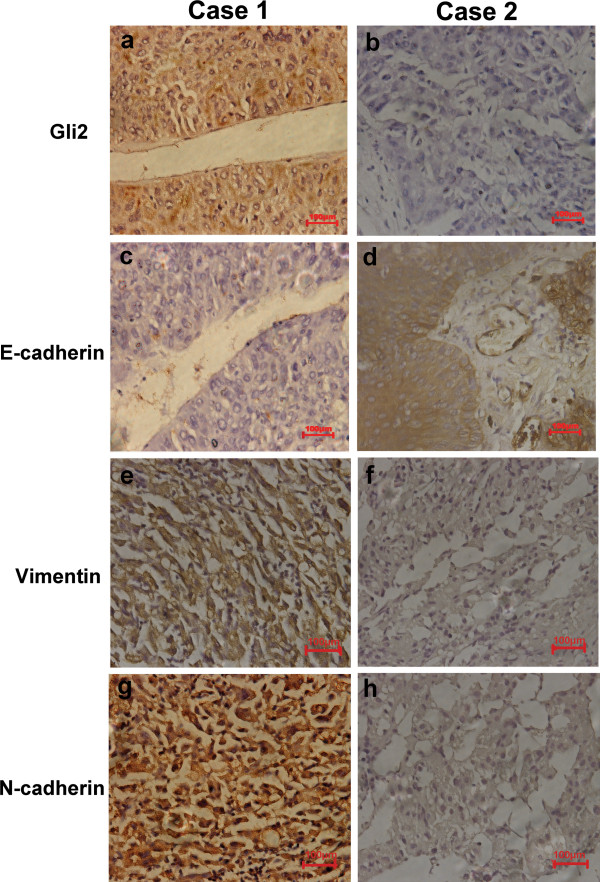
**Immunohistochemical staining of Gli2, E-cadherin, N-cadherin, and vimentin in two representative hepatocellular carcinoma (HCC) cases.** (**a,c,d,e)** Patient 1. Expression of Gli2, N-cadherin and vimentin was positive, whereas E-cadherin was negative. **(b,d,f,h)** Patient 2. Expression of Gli2, N-cadherin and vimentin was negative, whereas E-cadherin expression was positive. Original magnification × 400.

Given the role of Hedgehog signaling in inducing EMT through multiple regulators, such as Snail, ZEB1, ZEB2, Twist1, Twist2, and FOXC2, [17] we analyzed the relationship between Gli2 expression and that of E-cadherin, N-cadherin and vimentin in the 68 HCC samples. Expression of Gli2 was negatively correlated with E-cadherin, and positively correlated with N-cadherin and vimentin. Tissue with high expression levels for Gli2 also had high expression levels for N-cadherin (*r* = −0.505, *P* < 0.05) and vimentin (*r* = −0.468, *P* < 0.05), but low expression of E-cadherin (*r* = −0.302, *P* < 0.05) d Representative images are shown in Figure 2 and the statistical results are listed in Table 3.

**Table 3:** Association between expression of Gli2 and expressin of E-cadherin, N-cadherin and vimentin in patients with hepatocellular carcinoma

**Expression level**	**Gli2 expression**		**χ**^**2**^	***P *value**	**Association coefficient (*r*)**
	**High**	**Low**			
E-cadherin					
High	12	31	6.801	0.009^*^	−0.302
Low	15	10			
Vimentin					
High	35	8	6.801	0.009^*^	0.468
Low	7	18			
N-cadherin					
High	37	6	23.324	0.001^*^	−0.505
Low	7	18			

* *P* < 0.05.

### Survival analysis

**Figure 3:**
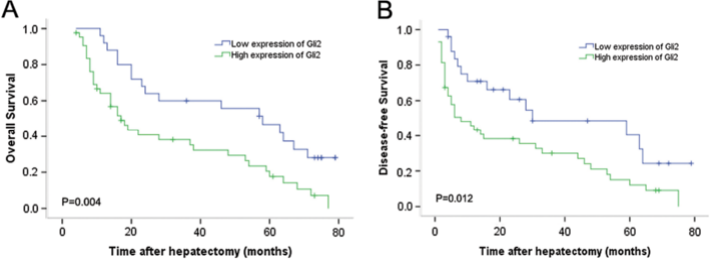
**Overall survival (OS) curves of patients with hepatocellular carcinoma (HCC) undergoing hepatectomy were compared between the groups with high and low expression of Gli2.** (**A**) OS; (**B**) disease-free survival.

We assessed the Kaplan-Meier estimates for the group with high Gli2 expression and the group with low Gli2 expression. Our results indicated that the median OS for the two groups were 17 and 58 months, respectively. The log-rank test showed as significant difference between the two survival curves; the OS rate of the group with high Gli2 expression was lower than that of the group with low Gli2 expression (*P* < 0.05). Similarly, the median DFS time for the two groups was 8 and 30 months, respectively, and patients with high Gli2 expression of Gli2 had a significantly shorter DFS compared with the patients with low Gli2 expression (*P* < 0.05) (Figure 3).

### Univariate and multivariate analyses for the prognostic value of Gli2 expression

In univariate analysis, the factors significantly associated with OS were tumor size, TNM stage, differentiation, tumor encapsulation, vascular invasion, and Gli2 expression, and those significantly associated with DFS were tumor size, tumor encapsulation, vascular invasion, and Gli2 expression (Table 4). Furthermore, multivariate Cox proportional hazards regression analysis indicated that in patients with HCC, tumor size, vascular invasion, and Gli2 expression were independent prognostic factors for OS, while tumor encapsulation, vascular invasion, and Gli2 expression were independent predictors for DFS (*P* < 0.05) (Table 5).

**Table 4:** Univariate analysis of factors associated with overall survival (OS) and disease-free survival (DFS) of 68 patients with hepatocellular carcinoma

**Factor**	***P *value**	
	**OS**	**DFS**
Gender (male versus female)	0.564	0.867
Age (≤60 versus >60 years)	0.430	0.682
Virus (HBV versus HCV and none)	0.410	0.247
AFP (≤200 versus >200 ng/ml)	0.909	0.626
Cirrhosis (absent versus present)	0.313	0.551
Tumor size (≤50 versus >50 mm)	0.005 ^*^	0.001 ^*^
TNM stage (I to II versus III to IV)	0.034 ^*^	0.056
Differentiation (well and moderate versus poor)	0.011 ^*^	0.110
Tumor number (solitary versus multiple)	0.401	0.741
Tumor encapsulation (intact versus absent or not intact)	0.011 ^*^	0.009 ^*^
Vascular invasion (absent versus present)	0.003 ^*^	0.001 ^*^
Gli2 expression (low versus high)	0.005 ^*^	0.017 ^*^

Abbreviations: AFP, alpha fetoprotein; HBV, hepatitis B virus; HCV, hepatitis C virus; TMN, tumor, node, metastasis.

* *P* < 0.05.

**Table 5:** Multivariate Cox regression analysis

**Factor**	***P *value**	**Hazard ratio**	**95% Confidence interval**
Overall survival			
Tumor size	0.002^*^	2.509	1.414 to 4.454
TNM stage	NS	—	—
Differentiation	NS	—	—
Tumor encapsulation	NS	—	—
Vascular invasion	0.004^*^	2.305	1.304 to 4.073
Gli2 expression	0.007^*^	2.239	1.244 to 4.028
Disease-free survival			
Tumor size	NS	—	—
Tumor encapsulation	0.001^*^	2.763	1.534 to 4.977
Vascular invasion	0.002^*^	2.516	1.397 to 4.533
Gli2 expression	0.042^*^	1.917	1.025 to 3.584

Abbreviations: AFP, alpha fetoprotein; NS, not significant.

* *P* < 0.05.

## Discussion

Gli2, a transcription factor of the Hh pathway, regulates expression of downstream target genes, including Gli1, Bcl-2, c-FLIP, cyclin D1, c-Myc and vascular endothelial growth factor (VEGF) [32-35], which has previously been implicated in the development of various human tumors, such as medulloblastomas, basal cell carcinoma, prostate and breast cancer, and HCC [28,36-38], Gli2 was reportedly overexpressed and related to poor survival of patients with pediatric medulloblastoma. Although high expression levels of the Gli2 protein has been reported in HCC cell lines and tissues [25,26,39], the association between Gli2 expression and prognosis in patients with HCC has not been elucidated. To analyze the role of Gli2 in HCC, we carried out immunohistochemical staining and found higher levels of Gli2 protein in HCC tissues compared with PLT and NLT.

Analyzing the association of Gli2 expression with pathologic characteristics in 68 patients with HCC identified a significant correlation of Gli2 expression with tumor differentiation, encapsulation, vascular invasion, early recurrence, and intra-hepatic metastasis. Kaplan-Meier analysis showed that patients with HCC who had high expression of Gli2 had significantly worse prognosis than those with low expression, both for OS and DFS. Multivariate analysis showed that Gli2 was an independent prognostic factor for both recurrence and survival in patients with HCC after hepatectomy. Meanwhile, tumor size and vascular invasion were also independent prognostic factors for OS, while tumor encapsulation and vascular invasion were independent prognostic factors for DFS. Therefore, the level of protein expression of Gli2, a novel molecular biomarker, may be a powerful prognostic indicator for recurrence and survival of patients with HCC.

Our results showed that high expression of Gli2 was associated with vascular invasion, early recurrence, and intra-hepatic metastasis in patients with HCC, suggesting that overexpression of Gli2 contributes to progression of HCC. Furthermore, the immunohistochemical results showed a significantly negative correlation between Gli2 and E-cadherin expression and a significantly positive correlation between Gli2 expression and both vimentin and N-cadherin expression. We speculate that Gli2 could play an important role in invasion and metastasis of HCC by inducing EMT.

In previous studies, EMT was characterized by decreased cell adhesion and increased motility, which was accompanied by downregulation of E-cadherin and upregulation of vimentin and N-cadherin [40,41]. Zheng *et al*. found that Gli1 protein expression was positively correlated with Shh and S100a4, and negatively correlated with E-cadherin [42]. EMT is regulated by several transcription factors, including Snail, ZEB1, ZEB2, Twist1, and Twist2, each of which bind to the E-cadherin promoter region and repress its transcription [43-49]. The Hh pathway induces JAG2 upregulation for Notch-CSL-mediated Snal1 upregulation, and also induces transforming growth factor (TGF)-β secretion for ZEB1 and ZEB2 upregulation via the TGF-β receptor and nuclear factor (NF)-κB. TGF-β-mediated downregulation of the microRNAs R-141, 200a, 200b, 200c, 205, and 429 results in upregulation of the ZEB1 and ZEB2 proteins. Activation of Hh signaling indirectly leads to EMT through Notch, TGF-β signaling cascades, and regulatory networks of microRNA [17]. Recently, Alexaki *et al*. investigated the role of Gli2 in the invasion and metastasis of melanoma and found that increased expression of Gli2 was associated in melanoma cell lines with loss of E-cadherin expression and increased capacity to invade a protein gel (Matrigel) and to form bone metastases in mice [50]. Taken together, these results indicate that Gli2 might lead to the increased early recurrence and development of intra-hepatic metastases of HCC by induction of EMT.

## Conclusion

Our current findings indicate for the first time that the expression level of Gli2 is high in HCC tissue, and this high expression shows a significant association with poor clinical outcome after hepatectomy and a more aggressive tumor phenotype because it induces EMT changes. However, further studies are needed to investigate the biochemical mechanisms by which Gli2 induces EMT of HCC.

## Competing interests

No conflict of interests to declare.

## Authors’ contributions

Study conception and design: DZ and PX. Drafting of manuscript: LC. Acquisition of data: YL. Surgical procedures and management of the patients: HL. Analysis and interpretation of data: XY. All authors read and approved the final manuscript.
